# Production of Hyaluronic Acid by *Streptococcus zooepidemicus* on Protein Substrates Obtained from *Scyliorhinus canicula* Discards

**DOI:** 10.3390/md13106537

**Published:** 2015-10-23

**Authors:** José A. Vázquez, Lorenzo Pastrana, Carmen Piñeiro, José A. Teixeira, Ricardo I. Pérez-Martín, Isabel R. Amado

**Affiliations:** 1 Grupo de Reciclado y Valorización de Materiales Residuales (REVAL), Instituto de Investigacións Mariñas (IIM-CSIC), r/Eduardo Cabello, 6. Vigo-36208 Galicia, Spain; 2 Departamento de Química Analítica y Alimentaria, Facultad de Ciencias de Ourense (Universidad de Vigo), Campus As Lagoas s/n, Ourense-32004 Galicia, Spain; E-Mails: pastrana@uvigo.es (L.P.); sabelara@uvigo.es (I.R.A.); 3 Servicio de Instrumentación Científica (SICIM), Instituto de Investigacións Mariñas (IIM-CSIC), r/Eduardo Cabello, 6. Vigo-36208 Galicia, Spain; E-Mail: cpineiro@iim.csic.es; 4 Centre of Biological Engineering, University of Minho, Campus Gualtar, 4710-057 Braga, Portugal; E-Mail: jateixeira@deb.uminho.pt; 5 Grupo de Bioquímica de Alimentos, Instituto de Investigacións Mariñas (IIM-CSIC), r/Eduardo Cabello, 6. Vigo-36208 Galicia, Spain; E-Mail: ricardo@iim.csic.es

**Keywords:** viscera waste valorization, hyaluronic acid production, *Streptococcus zooepidemicus*, marine peptones, *Scyliorhinus canicula* by-products, logistic equation

## Abstract

This work investigates the production of hyaluronic acid (H) by *Streptococcus equi* subsp. *zooepidemicus* in complex media formulated with peptones obtained from *Scyliorhinus canicula* viscera by-products. Initially, in batch cultures, the greatest productions were achieved using commercial media (3.03 g/L) followed by peptones from alcalase hydrolyzed viscera (2.32 g/L) and peptones from non-hydrolyzed viscera (2.26 g/L). An increase of between 12% and 15% was found in subsequent fed-batch cultures performed on waste peptones. Such organic nitrogen sources were shown to be an excellent low-cost substrate for microbial H, saving more than 50% of the nutrient costs.

## 1. Introduction

One of the most important macromolecules for cosmetic and pharmacological formulations is hyaluronic acid (H). It is a high-molecular-mass glycosaminoglycan polysaccharide that can be found in different animal tissues such as skin, cartilage, vitreous humor, synovial liquid, bacterial cell capsule, *etc.* [1,2,3]. Its rheological and biological features including pseudoplasticity, biocompatibility material, and water holding capacity are especially adequate and essential for multiple types of commercial applications in different fields [4,5,6]. Although the production of H has been, during past decades, based on its extraction from bovine vitreous humor, umbilical cord, or rooster comb, microbial H from *Streptococcus* strains is currently the main alternative. Marine sources of H, such as fish vitreous humor, have also been explored, but lower yields than from the microbial sources were found [7,8].

Nevertheless, the cost of H production is basically dependent on the price of the commercial culture media. Streptococci are facultative anaerobe bacteria that show fastidious nutrient requirements in relation to the organic nitrogen [9,10] which cannot be replaced by inorganic nitrogen salts. The formulation of low-cost media is therefore essential for the industrial production of H. In this context, peptones generated from fishing by-products and discards are valuable and effective substrates [11]. In addition, the new policy of zero discards and the exhaustive reduction of marine wastes by the European Union (EU) requires the use of valorizing solutions to solve them. This microbial bioconversion strategy is also framed into the biorefinery concept which is one of the most important pillars to develop in the next decade from the blue biotechnology issue.

The small-spotted catshark (*Scyliorhinus canicula*) is an abundant fish in the northeastern Atlantic Ocean and Mediterranean Sea which is considered as a discard in some fisheries [12,13]. Recently, the processes for the recovery and purification of another glycosaminoglycan (chondroitin sulfate) from cartilage by-products of this species have been optimized [14]. However, to our knowledge, no valorized alternatives for viscera wastes have yet been developed. The present work studies the feasibility of peptones obtained from three treatments of *S. canicula* viscera as nitrogen sources for H production by *Streptococcus equi* subsp. *zooepidemicus* ATCC 35246.

## 2. Results and Discussion

**Figure 1:**
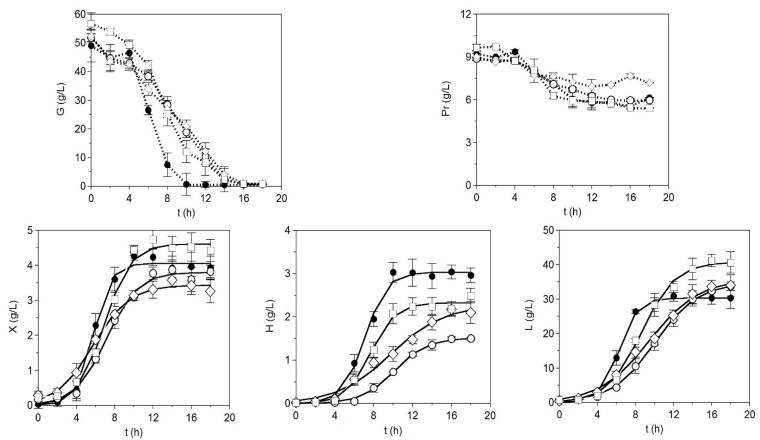
Batch cultivations of *S. zooepidemicus* on different nutritive media. G: glucose, Pr: total proteins, X: biomass, H: hyaluronic acid, L: lactic acid. ◊: Medium A, ○: Medium B, □: Medium C, ●: CM. In the case of bioproductions (X, L, and H), experimental data were fitted to the logistic Equation (1) (continuous line). Error bars are the confidence intervals (*n* = 2, α = 0.05).

In order to evaluate the viability of the three nitrogen sources obtained from small-spotted catshark wastes, commercial tryptone in the control medium was substituted by the peptones prepared using thermal autohydrolysis and enzyme catalysis of viscera. The remaining nutrients were similar in all media. Time-courses of batch cultures are displayed in Figure 1. The experimental data of the bioproductions quantified (biomass: X, lactic acid: L, and hyaluronic acid: H) were modeled on the logistic Equation (1). The agreement between predicted and experimental data was excellent (Table 1), with values of *R*^2^ higher than 0.986. The *p*-values from Fisher’s *F*-test also indicated the consistency of model (1) to describe the cultivation data.

All the kinetic parameters defined by Equations (1)–(4) for the three bioproductions were statistically significant (Student’s *t*-test, Table 1). According to these parameters, the highest maximum biomass (*X_m_* = 4.61 ± 0.26 g/L) and lactic acid production (*L_m_* = 40.74 ± 1.82 g/L) were found in Medium C, but CM medium conducted the highest maximum hyaluronic acid production (*H_m_* = 3.03 ± 0.14 g/L) and the maximum and specific maximum rate for X, L, and H productions (*v_x_*, *v_h_*, *v_l_*, *μ_x_*, *μ_h_*, and *μ_l_*). In concordance, the times to reach the asymptotic or plateau phase of sigmoid trends in CM (*t_P_*) were shorter than in alternative media. The production of the glycosaminoglycan, taking into account the values of *H_m_*, followed this sequence between broths: CM > Medium C = Medium A > Medium B. The *H_m_* results in Media C and A were somewhat inferior (2.32 ± 0.13 g/L) to the tryptone ones, but were nevertheless remarkable and similar to those derived from tuna viscera (2.41 ± 0.02 g/L) [15]. Although the slight and not significant difference between the H productions in Media A and C indicates that the use of alcalase in the process of viscera hydrolysis could be excluded, the greater concentration of protein extracted by means of enzyme application could justify this.

**Table 1:** Parametric estimations corresponding to the logistic Equation (1) applied to *S. zooepidemicus* batch cultures. Numerical values of the parameters are shown with their confidence intervals. *R*^2^ is the determination coefficients for the mathematical fittings to Equation (1) and *p*-values from Fisher’s *F*-test verify the robustness of the equation. The different production yields are also calculated.

Parameters	Medium A	Medium B	Medium C	CM
*X_m_* (g/L)	3.43 ± 0.15	3.80 ± 0.20	4.61 ± 0.26	4.05 ± 0.16
*v_x_* (g·L^−1^·h^−1^)	0.461 ± 0.009	0.587 ± 0.131	0.835 ± 0.230	1.11 ± 0.33
*λ_x_* (h)	2.03 ± 0.77	3.92 ± 0.81	4.15 ± 0.86	3.99 ± 0.62
*τ_x_* (h)	5.75 ± 0.44	7.15 ± 0.47	6.91 ± 0.48	5.80 ± 0.28
*μ_x_* (h^−1^)	0.538 ± 0.109	0.618 ± 0.151	0.725 ± 0.488	1.10 ± 0.32
*t_x_* (h)	9.46 ± 0.80	10.39 ± 0.67	9.66 ± 0.71	7.62 ± 0.34
*Y_X/G_*	0.058	0.071	0.075	0.080
*Y_X/Pr_*	1.632	1.254	0.991	1.275
*R*^2^	0.994	0.994	0.993	0.995
*p*-Value	<0.001	<0.001	<0.001	<0.001
*H_m_* (g/L)	2.26 ± 0.33	1.51 ± 0.06	2.32 ± 0.13	3.03 ± 0.14
*v_h_* (g·L^−1^·h^−1^)	0.207 ± 0.053	0.225 ± 0.028	0.456 ± 0.126	0.667 ± 0.169
*λ_h_* (h)	4.23 ± 1.46	6.79 ± 0.45	5.14 ± 0.79	4.81 ± 0.66
*τ_h_* (h)	9.69 ± 1.33	10.15 ± 0.29	7.68 ± 0.44	7.09 ± 0.36
*μ_h_* (h^−1^)	0.366 ± 0.128	0.596 ± 0.085	0.785 ± 0.232	0.879 ± 0.235
*t_h_* (h)	15.15 ± 2.01	13.50 ± 0.56	10.23 ± 0.89	9.36 ± 0.40
*Y_H/G_*	0.040	0.029	0.044	0.061
*Y_H/Pr_*	1.143	0.512	0.584	0.967
*Y_H/X_*	0.700	0.408	0.589	0.759
*R*^2^	0.986	0.998	0.993	0.995
*p*-Value	<0.001	<0.001	<0.001	<0.001
*L_m_* (g/L)	35.78 ± 2.28	34.60 ± 1.14	40.74 ± 1.82	30.25 ± 0.77
*v_l_* (g·L^−1^·h^−1^)	3.46 ± 0.54	3.90 ± 0.29	5.73 ± 0.89	7.81 ± 1.39
*λ_l_* (h)	4.31 ± 0.84	5.61 ± 0.35	5.09 ± 0.61	4.34 ± 0.38
*τ_l_* (h)	9.48 ± 0.71	10.05 ± 0.27	8.65 ± 0.38	6.28 ± 0.20
*μ_l_* (h^−1^)	0.387 ± 0.079	0.451 ± 0.042	0.562 ± 0.099	1.03 ± 0.19
*t_l_* (h)	14.65 ± 1.34	14.48 ± 0.59	12.21 ± 0.70	8.21 ± 0.25
*Y_L/G_*	0.651	0.643	0.713	0.622
*Y_L/Pr_*	18.44	11.29	9.42	9.87
*R*^2^	0.995	0.999	0.997	0.998
*p*-Value	<0.001	<0.001	<0.001	<0.001

The consumption of glucose was faster in CM and the protein time-course was similar in Media B, C, and CM. The final protein uptake was inferior in Medium A. The best productivities in relation to protein uptake were also generated by Medium A. In the remaining cases, the highest yields were mainly defined by CM. The molecular weights of H sampled at the end of the fermentations (18 h) were 1800, 1730, 1900, and 1850 kDa for Media A, B, C, and CM, respectively.

The concentrations of H and corresponding molecular weights obtained with the bacteria *S. zooepidemicus* ATCC 35246 in the present study were similar or, in some cases, higher than those reported in the literature [10,16]. Anaerobic and aerobic cultures in a base medium formulated with glucose, mineral salts (MgSO_4_ and Na_2_HPO_4_), and yeast extract as unique sources of nitrogen showed productions around 1.5 and 2.5 g/L of H, respectively [17]. The molecular weights of such H were of 2 and 3 MDa, respectively. These authors also found values of *μ_m_* (0.45–0.75 h^−1^), working under different experimental conditions, in agreement with the outcomes in the alternative media (Table 1). However, our yields of hyaluronic production/glucose consumption were lower in comparison with those previously reported in the mentioned work [17]. In this context, Johns *et al.* [16] previously established the best conditions of agitation (>400 rpm) and pH (6.6–6.9) for *S. zooepidemicus* ATCC 35246 in a complex medium (similar to the base medium supplemented with guanine, adenine, and uracil). The values of H and L productions were similar to those described herein.

In recent times, some culture media based on nutrients recovered from agrifood wastes have been studied as new options for the bacterial production of H. Fruit bagasse (cashew apple) was studied as a substrate for *S. zooepidemicus* fermentations in a solid-state modality generating 6 mg/g of H [18]. Low levels of H (~0.2 g/L) were achieved when whey or soy protein were utilized as nitrogen sources in Erlenmeyer flask experiments [19,20]. In no case were such bioproductions comparable to those obtained with marine peptones, mainly because batch cultures were performed at flask scale and without pH control. It is well known that the lack of constant pH conditions (among 6.0–7.0) and the subsequent spontaneous evolution of the acidic environment in *S. zooepidemicus* culture provoke the inhibition of H production [16,17,21].

**Figure 2:**
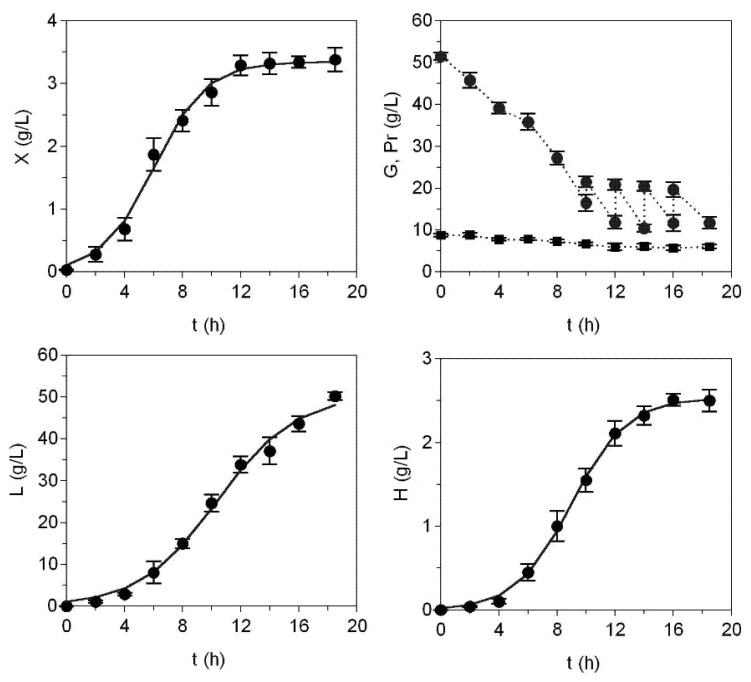
Fed-batch cultivation of *S. zooepidemicus* on medium formulated with peptones obtained from *S. canicula* viscera without hydrolysis (Medium A). Concentrated glucose solutions were employed for fed-batch operatory. Experimental data of bioproductions were fitted to the logistic Equation (1) (continuous line). Error bars are the confidence intervals (*n* = 2, α = 0.05).

Selecting the best residual media (A and C), fed-batch cultures were performed in order to increase the biosynthesis production of the polysaccharide by *S. zooepidemicus*. Figure 2 shows the case of Medium A which included a glucose fed-batch every 2 h from 10 h of culture. The numerical analyses of all cultures are summarized in Table 2. Logistic Equation (1) was again a perfect tool for kinetics modeling (*R*^2^ > 0.978 and *p* < 0.001). CM was the most effective medium in terms of maximum concentration bioproductions and rates of productions. However, the increment of *H_m_* comparing batch and fed-batch resources was (in %) 12, 15, and 7 for Media A, C, and CM, respectively. Moreover, the highest yields of biomass, hyaluronic, and lactic acids as functions of protein consumed were defined by Medium A. The *M_w_* of the biopolymer in that medium was larger (2110 kDa) than previously detected in the batch.

These outcomes of hyaluronic acid formation on residual media were in agreement with those previously found using peptones extracted from *Isirus oxyrhinchus* and *Raja clavata* by-products [11]. However, the clear biphasic sigmoid patterns described in these fed-batch cultivations were not observed in the current study. Other authors have also investigated the capacity of the fed-batch strategy to increase the microbial hyaluronic acid production [22] or to perform successive batch fermentations [23]. In general, lactic acid bacteria have also shown excellent responses to the glucose-intermittent fed in terms of enhancing cultivation time and biomass, and lactic acid and bacteriocin productions [24,25,26].

**Figure 3:**
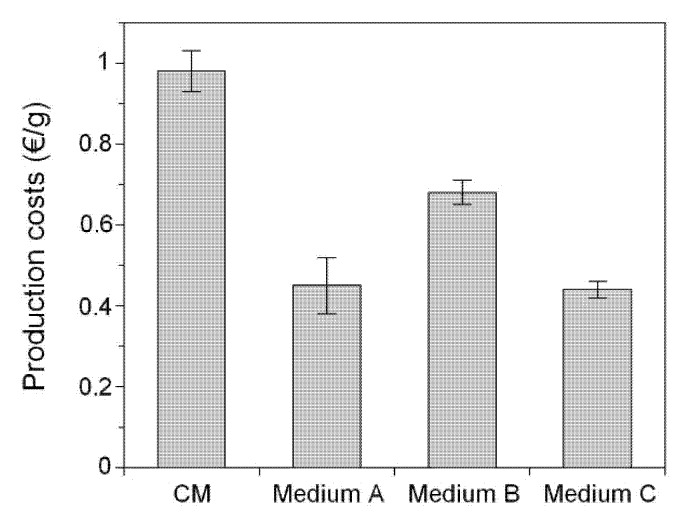
Hyaluronic production costs in each medium tested for batch culture conditions. Error bars are the confidence intervals (*n* = 2, α = 0.05).

Based on the commercial prices of the tryptone, yeast extract glucose, and mineral salts ingredients, the costs of H production in the Media A, B, C, and CM were calculated (Figure 3). The *H_m_* data used for calculation were selected from Table 1. The three alternative peptones proved to be an excellent source of nitrogen in terms of reducing cost. Media B, A, and C reduced the price of a gram of hyaluronic acid produced in batch fermentations by 31%, 54%, and 55%, respectively, in comparison with CM. Similar cost reductions were observed in fed-batch cultures (data not shown). In addition, current results were concordant with those reported when medium for *S. zooepidemicus* was formulated with tuna peptone and mussel-processing effluents [15].

**Table 2:** Parametric estimations corresponding to the logistic Equation (1) applied to *S. zooepidemicus* fed-batch cultures. Numerical values of the parameters are shown with their confidence intervals. *R*^2^ is the determination coefficient for the mathematical fittings to Equation (1) and *p*-values from Fisher’s *F*-test verify the robustness of the equation. The different production yields are also calculated.

Parameters	Medium A	Medium C	CM
*X_m_* (g/L)	3.34 ± 0.16	3.27 ± 0.17	4.56 ± 0.29
*v_x_* (g·L^−1^·h^−1^)	0.467 ± 0.098	0.464 ± 0.103	0.930 ± 0.350
*λ_x_* (h)	2.47 ± 0.83	2.69 ± 0.87	3.46 ± 1.03
*τ_x_* (h)	6.05 ± 0.48	6.22 ± 0.50	8.34 ± 0.77
*μ_x_* (h^−1^)	0.559 ± 0.128	0.567 ± 0.138	0.820 ± 0.321
*t_x_* (h)	9.63 ± 0.60	9.74 ± 0.87	7.62 ± 0.34
*Y_X/G_*	0.047	0.049	0.052
*Y_X/Pr_*	1.201	0.976	1.027
*R*^2^	0.994	0.994	0.988
*p*-Value	<0.001	<0.001	<0.001
*H_m_* (g/L)	2.53 ± 0.09	2.66 ± 0.17	3.23 ± 0.25
*v_h_* (g L^−1^·h^−1^)	0.332 ± 0.035	0.321 ± 0.054	0.589 ± 0.234
*λ_h_* (h)	5.19 ± 0.44	5.42 ± 0.75	3.76 ± 1.22
*τ_h_* (h)	9.00 ± 0.27	9.56 ± 0.52	6.50 ± 0.65
*μ_h_* (h^−1^)	0.526 ± 0.061	0.483 ± 0.095	0.730 ± 0.299
*t_h_* (h)	12.80 ± 0.99	13.70 ± 0.61	9.24 ± 0.81
*Y_H/G_*	0.035	0.039	0.039
*Y_H/Pr_*	0.896	0.772	0.767
*Y_H/X_*	0.746	0.791	0.747
*R*^2^	0.999	0.996	0.986
*p*-Value	<0.001	<0.001	<0.001
*L_m_* (g/L)	50.50 ± 5.44	51.13 ± 4.16	81.88 ± 19.65
*v_l_* (g·L^−1^·h^−1^)	4.68 ± 0.83	5.08 ± 0.76	6.53 ± 1.92
*λ_l_* (h)	5.01 ± 0.99	5.11 ± 0.78	4.49 ± 1.89
*τ_l_* (h)	10.42 ± 0.94	10.14 ± 0.69	10.76 ± 2.22
*μ_l_* (h^−1^)	0.370 ± 0.090	0.398 ± 0.079	0.319 ± 0.143
*t_l_* (h)	15.82 ± 2.79	15.16 ± 2.19	17.03 ± 4.01
*Y_L/G_*	0.700	0.762	0.895
*Y_L/Pr_*	17.99	15.04	17.77
*R*^2^	0.994	0.996	0.978
*p*-Value	<0.001	<0.001	<0.001

## 3. Experimental Section

### 3.1. Preparation of Protein Substrate (Peptones)

*S. canicula* by-products (stomachs and intestines) were collected from fish-processing industries located in Vigo (Spain). Fresh material was mixed with distilled water (40% w/v), grinded, and divided to perform the following procedures: (a) autoclaving at 101 °C/1 h, centrifugation at 12,000× *g*/30 min, and supernatant recovery (Peptone A); (b) autohydrolysis of homogenate by incubation at 30 °C/200 rpm/5 h, autoclaving of the autohydrolysate at 101 °C/1 h, centrifugation at 12,000× *g*/30 min, and supernatant recovery (Peptone B); (c) enzymatic hydrolysis by alcalase addition (2% v/p, 48 AU/kg) and incubation at 50 °C/200 rpm/3 h, autoclaving of the enzymatic hydrolysate at 101 °C/1 h, centrifugation at 12,000× *g*/30 min, and supernatant recovery (Peptone C). The composition of peptones was (in g/L): 15.3 ± 0.2 of protein and 1.8 ± 0.1 of reducing sugars (Peptone A), 15.5 ± 0.3 of protein and 2.4 ± 0.3 of reducing sugars (Peptone B), 23.5 ± 0.4 g/L of protein and 2.2 ± 0.2 of reducing sugars (Peptone C).

### 3.2. Bacterial Strain

Growth experiments and hyaluronic acid production were carried out using the Lancefield group C, β-hemolytic and mucoid bacterium: *Streptococcus equi* subsp. *zooepidemicus* ATCC 35246 obtained from the American Type Culture Collection (Manassas, VA, USA). Stocks of this bacterium were stored at −80 °C in complex medium (defined below) with 25% glycerol. Inocula were conducted according the steps reported in the previous work [11].

### 3.3. Microbiological Culture Media and Growth Conditions

The complex (CM) and alternative growth media for *S. zooepidemicus* contained the following nutrients (in g/L of distilled water): glucose, 50; yeast extract, 5; K_2_HPO_4_, 2.0; KH_2_PO_4_, 2.0; MgSO_4_, 0.5; (NH_4_)SO_4_, 0.5; and either tryptone (Cultimed, Panreac Química, Spain), 15, or the peptones (A, B, C) obtained from each *S. canicula* visceral treatment at the protein-Lowry concentration of 8. These media were: Medium A, Medium B, and Medium C formulated with Peptone A, Peptone B, and Peptone C, respectively. The pH in all cultures was adjusted to 6.7 after autoclaving and sterilized at 121 °C/15 min.

*S. zooepidemicus* was cultivated in a glass 2 L bioreactor (with a working volume of 1.8 L) at 37 °C, 500 rpm of agitation, without aeration, and pH controlled with sterile solutions of NaOH and HCl (both 5 M). In the fed-batch cultivations, glucose was added every 2 h (from 8 to 10 h of culture) up to a concentration of 20 g/L using a sterile glucose solution of 500 g/L.

### 3.4. Analytical Methods

The procedures of sampling from the bioreactor, preparation of samples, and purification of H are extensively described in a previous report [15]. Briefly, samples from the bioreactor were initially blended with a 10% volume of 5% (w/v) SDS for 10 min and centrifuged at 5000× *g*/30 min. H was precipitated by mixing the supernatant with three volumes of ethanol and then centrifuged at 5000× *g*/10 min. The sediment was redissolved with one volume of NaCl (1.5 M) and three volumes of ethanol and subsequently centrifuged again at 5000× *g*/10 min. Finally, this last sediment was resuspended in distilled water for HA determination. The rest of the determinations performed (in duplicate) were: (1) optical density (OD) at 700 nm and subsequent calculation of biomass (dry weight) using a corresponding calibration curve; (2) total soluble proteins according the method of Lowry *et al.* [27]; (3) H analyzed using the method of Blumenkrantz and Asboe-Hansen [28] with the modifications of Murado *et al.* [29]; (4) reducing sugars measured by the 3,5-dinitrosalicylic reaction [30]; (5) glucose, lactic acid, and molecular weight (Mw) of H using HPLC methodology [11]. Thus, ION-300 column (Transgenomic, San José, CA, USA) with 6 mM H_2_SO_4_ as mobile phase (flow = 0.4 mL/min) at 65 °C and a refractive-index detector were used for lactic acid and glucose quantification. Mw of H was determined by size-exclusion chromatography on HPLC equipped with an Ultrahydrogel linear column (Waters, Milford, MA, USA) using 0.1 M NaNO_3_ as mobile phase (flow = 0.6 mL/min) and a refractive-index detector. Standards of dextran (Sigma, St. Louis, MO, USA) with different molecular weights (5, 12, 25, 50, 80, 150, 270, 410, 670, 1400, 2000, and 3500 kDa) were used for calibration.

### 3.5. Modeling of S. zooepidemicus Cultivation

The logistic equation was chosen to model the kinetic profiles of the different bioproductions (P) as biomass (X), hyaluronic acid (H), and lactic acid (L) productions [31]:

(1) $$P=\frac{P_{m}}{1+\exp\left[2+\frac{4v_{P}\left(\lambda_{P}-t\right)}{P_{m}}\right]}$$

Complementarily, we have calculated other parameters with clear biological means to help the characterization of the cultures in the media studied:

(2) $$\mu_{P}=\;\frac{4v_{P}}{P_{m}}$$

(3) $$\tau_{P}=\lambda_{P}+\frac{2}{\mu_{P}}$$

(4) $$t_{mP}=\;\tau_{P}+\;\frac{P_{m}}{2v_{P}}$$

where *P* is the product determined (*X*, *L*, or *H*); *P_m_* is the maximum product formation (g/L); *v_P_* is the maximum production rate (g·L^−1^·h^−1^); *λ_P_* is the product’s lag phase (h); *μ_P_* is the specific maximum production rate (h^−1^); *τ_P_* is the time required to achieve half the maximum production (h); and *t_mP_* is the time required to reach the maximum production (h). In addition, the yields of productions on glucose (*Y_P_*/*Y_G_*), protein uptakes (*Y_P_*/*Y_Pr_*), and hyaluronic acid relative to biomass (*Y_H_*/*Y_X_*) were also determined.

### 3.6. Numerical and Statistical Analyses

Fitting procedures and parametric estimations calculated from the results were carried out by minimizing the sum of quadratic differences between the observed and model-predicted values, using the non-linear least-squares (quasi-Newton) method provided by the macro-“Solver” of the Microsoft Excel spreadsheet. Confidence intervals from the parametric estimates (Student’s *t*-test) and consistence of mathematical models (Fisher’s *F*-test) were assessed by “SolverAid” macro (Levie’s Excellaneous website: http://www.bowdoin.edu/~rdelevie/excellaneous).

## 4. Conclusions

Protein material derived from fishing discards (*S. canicula* viscera) proved to be an appropriate alternative to replace commercial tryptone in the microbial production of H. Peptones obtained by alcalase and thermal treatment of by-products yielded more than 2 g/L of the polysaccharide with higher than 1800 kDa in batch fermentations. Furthermore, fed-batch operatory significantly improved the productions but not the molecular weights of H. In all cases, the reduction of the prime cost of H using marine-based media was remarkable.
